# United States Centers for Disease Control and Prevention support for influenza surveillance, 2013–2021

**DOI:** 10.2471/BLT.21.287253

**Published:** 2022-04-03

**Authors:** Margaret McCarron, Rebecca Kondor, Kinda Zureick, Chelsey Griffin, Christian Fuster, Aspen Hammond, Maja Lievre, Katelijn Vandemaele, Joseph Bresee, Xiyan Xu, Vivien G Dugan, Vashonia Weatherspoon, Thelma Williams, April Vance, Alicia M Fry, Magdi Samaan, Julia Fitzner, Wenqing Zhang, Ann Moen, David E Wentworth, Eduardo Azziz-Baumgartner

**Affiliations:** a Influenza Division, Centers for Disease Control and Prevention, 1600 Clifton Road NE MS A32, Atlanta, GA 30329, United States of America.; b Global Influenza Programme, World Health Organization, Geneva, Switzerland.

## Abstract

**Objective:**

To assess the stability of improvements in global respiratory virus surveillance in countries supported by the United States Centers for Disease Control and Prevention (CDC) after reductions in CDC funding and with the stress of the coronavirus disease 2019 (COVID-19) pandemic.

**Methods:**

We assessed whether national influenza surveillance systems of CDC-funded countries: (i) continued to analyse as many specimens between 2013 and 2021; (ii) participated in activities of the World Health Organization’s (WHO) Global Influenza Surveillance and Response System; (iii) tested enough specimens to detect rare events or signals of unusual activity; and (iv) demonstrated stability before and during the COVID-19 pandemic. We used CDC budget records and data from the WHO Global Influenza Surveillance and Response System.

**Findings:**

While CDC reduced per-country influenza funding by about 75% over 10 years, the number of specimens tested annually remained stable (mean 2261). Reporting varied substantially by country and transmission zone. Countries funded by CDC accounted for 71% (range 61–75%) of specimens included in WHO consultations on the composition of influenza virus vaccines. In 2019, only eight of the 17 transmission zones sent enough specimens to WHO collaborating centres before the vaccine composition meeting to reliably identify antigenic variants.

**Conclusion:**

Great progress has been made in the global understanding of influenza trends and seasonality. To optimize surveillance to identify atypical influenza viruses, and to integrate molecular testing, sequencing and reporting of severe acute respiratory syndrome coronavirus 2 into existing systems, funding must continue to support these efforts.

## Résumé

**Objectif:**

Évaluer la stabilité des améliorations en matière de surveillance mondiale des virus respiratoires dans les pays soutenus par les Centers for Disease Control and Prevention (CDC) des États-Unis d'Amérique, après les baisses de financement des CDC et avec le stress causé par la pandémie de maladie à coronavirus 2019 (COVID-19).

**Méthodes:**

Nous avons examiné les mécanismes nationaux de surveillance de la grippe dans les pays financés par les CDC pour déterminer: (i) s'ils continuaient à analyser le même nombre d'échantillons entre 2013 et 2021; (ii) s'ils participaient à des activités du Système mondial de surveillance de la grippe et de riposte mis en place par l'Organisation mondiale de la Santé (OMS); (iii) s'ils testaient suffisamment d'échantillons pour détecter des événements rares ou des signes d'activité inhabituelle; et enfin, (iv) s'ils faisaient preuve de stabilité avant et pendant la pandémie de maladie à coronavirus 2019 (COVID-19). Nous avons utilisé les documents budgétaires des CDC, ainsi que les données du Système mondial OMS de surveillance de la grippe et de riposte.

**Résultats:**

Bien que les CDC aient réduit d'environ 75% leur financement aux différents pays pour étudier la grippe au cours de la décennie écoulée, le nombre d'échantillons testés chaque année est resté stable (2261 en moyenne). Les signalements variaient fortement en fonction du pays et de la zone de transmission. Les pays financés par les CDC représentaient 71% (plage comprise entre 61% et 75%) des échantillons inclus dans les consultations de l'OMS sur la composition des vaccins contre les virus grippaux. En 2019, seulement huit des 17 zones de transmission ont envoyé suffisamment d'échantillons aux centres collaborant avec l'OMS pour identifier avec certitude les variants antigéniques avant la réunion sur la composition des vaccins.

**Conclusion:**

Des progrès considérables ont été réalisés en matière de compréhension des tendances et du caractère saisonnier de la grippe. Néanmoins, le financement doit se poursuivre afin d'améliorer la surveillance et, par conséquent, d'identifier les virus grippaux atypiques, mais aussi d'intégrer les tests moléculaires, le séquençage et le signalement du coronavirus 2 du syndrome respiratoire aigu sévère dans les systèmes existants.

## Resumen

**Objetivo:**

Valorar la estabilidad de las mejoras en la vigilancia mundial de los virus respiratorios en los países que reciben apoyo de los Centros para el Control y la Prevención de Enfermedades (CDC) de los Estados Unidos después de las reducciones en la financiación de los CDC y ante la situación de estrés por la pandemia de la enfermedad por coronavirus de 2019 (COVID-19).

**Métodos:**

Se analizó si los sistemas nacionales de vigilancia de la influenza de los países que reciben fondos de los CDC: i) seguían analizando la misma cantidad de muestras entre 2013 y 2021; ii) participaban en las actividades del Sistema Mundial de Vigilancia y Respuesta a la Gripe de la Organización Mundial de la Salud (OMS); iii) analizaban suficientes muestras para detectar acontecimientos raros o señales de actividad inusual; y iv) demostraban estabilidad antes y durante la pandemia de la enfermedad por coronavirus de 2019 (COVID-19). Se utilizaron los registros de presupuesto de los CDC y los datos del Sistema Mundial de Vigilancia y Respuesta a la Gripe de la OMS.

**Resultados:**

Aunque los CDC redujeron los fondos destinados a la gripe por país en casi un 75 % a lo largo de 10 años, el número de muestras analizadas al año se mantuvo estable (media de 2261). Los informes variaron de manera sustancial según el país y la zona de transmisión. Los países que recibieron financiación de los CDC representaron el 71 % (rango entre el 61 y el 75 %) de las muestras incluidas en las consultas de la OMS sobre la composición de las vacunas antigripales. En 2019, solo ocho de las 17 zonas de transmisión enviaron suficientes muestras a los centros colaboradores de la OMS antes de la reunión sobre la composición de la vacuna para identificar de una manera fiable las variantes antigénicas.

**Conclusión:**

Se ha logrado un gran progreso en la comprensión global de las tendencias y la estacionalidad de la gripe. Para optimizar la vigilancia con el fin de identificar los virus atípicos de la gripe e integrar las pruebas moleculares, la secuenciación y la notificación del coronavirus del síndrome respiratorio agudo grave de tipo 2 en los sistemas existentes, la financiación debe seguir apoyando estos esfuerzos.

## ملخص

الغرض تقييم استقرار التحسينات في المراقبة العالمية لفيروس الجهاز التنفسي في الدول التي تدعمها المراكز الأمريكية لمكافحة الأمراض والوقاية منها (CDC)، بعد التخفيضات في تمويل هذه المراكز، ومع ضغط جائحة مرض فيروس كورونا 2019 (كوفيد 19).

الطريقة قمنا بتقييم ما إذا كانت الأنظمة الوطنية لمراقبة الإنفلونزا في الدول التي تمولها المراكز الأمريكية لمكافحة الأمراض والوقاية منها: (1) واصلت تحليل العديد من العينات بين عامي 2013 و2021؛ و(2) شارك في أنشطة النظام العالمي لمراقبة الإنفلونزا والاستجابة التابع لمنظمة الصحة العالمية؛ و(3) فحصت عينات كافية لاكتشاف الأحداث والإشارات النادرة للنشاط غير العادي؛ و(4) أظهرت الاستقرار قبل وأثناء جائحة مرض فيروس كورونا 2019 (كوفيد 19). واستخدمنا سجلات وبيانات ميزانية مراكز CDC من النظام العالمي لمراقبة الإنفلونزا والاستجابة التابع لمنظمة الصحة العالمية.

النتائج بينما خفضت مراكز CDC تمويل الإنفلوانزا لكل دولة بنحو 75% على مدى 10 سنوات، فإن عدد العينات التي يتم فحصها سنويًا ظل مستقرًا (متوسط 2261). اختلفت التقارير بشكل كبير حسب الدولة ومنطقة الإرسال. إن الدول التي حصلت على تمويل من مراكز CDC شكلت 71% (المدى 61 إلى 75%) من العينات المتضمنة في استشارات منظمة الصحة العالمية حول تكوين لقاحات فيروس الإنفلونزا. في عام 2019، ثمانية فقط من مناطق الانتقال الـ 17 أرسلت عينات كافية إلى المراكز المتعاونة مع منظمة الصحة العالمية قبل اجتماع تكوين اللقاح لتحديد المتغيرات المستضدية بشكل موثوق.

الاستنتاج تم إحراز تقدم كبير في الفهم العالمي لاتجاهات ومواسم الأنفلونزا. لتحسين المراقبة لتحديد فيروسات الأنفلونزا غير المعتادة، ودمج الفحص الجزيئي، والتسلسل، والإبلاغ عن متلازمة الالتهاب التنفسي الحاد الوخيم فيروس كورونا 2 في الأنظمة الحالية، يجب أن يستمر التمويل في دعم هذه الجهود.

## 摘要

**目的:**

旨在评估由美国疾病控制和预防中心 (CDC) 支持的国家在 CDC 资金减少和新型冠状病毒肺炎大流行压力下的全球呼吸系统病毒监测改善情况的稳定性。

**方法:**

我们评估了 CDC 资助的国家的国家流感监测系统是否：(i) 继续尽可能多地分析 2013 至 2021 年间的样本；(ii) 参与了世界卫生组织 (WHO) 的全球流感监测和应对系统的活动；(iii) 测试了足够多的样本以检测小概率事件或异常活动信号；以及 (iv) 在新型冠状病毒肺炎大流行之前和期间表现出稳定性。我们使用了 CDC 预算记录和世卫组织全球流感监测和应对系统的数据。

**结果:**

虽然 CDC 在 10 年内将每个国家的流感基金减少了约 75%，但每年测试的样本数量保持稳定（平均 2261 份）。报告因国家和传播区而异。由 CDC 资助的国家的样本占世卫组织磋商的流感病毒疫苗组合物中包含的样本的 71%（范围为 61-75%）。在 2019 年，17 个传播区中仅八个区域在疫苗组合物大会之前向世卫组织合作中心发送了足够数量的样本，以供识别抗原变体。

**结论:**

全球对流感趋势和季节性的认识取得很大进展。要优化监测以识别非典型流感病毒，并将严重急性呼吸综合征冠状病毒 2 的分子检测、测序和报告整合到现有系统中，必须持续投入资金予以支持。

## Резюме

**Цель:**

Оценить стабильность улучшений в глобальном эпиднадзоре за респираторными вирусами в странах, поддерживаемых центрами США по контролю и профилактике заболеваний (CDC), после сокращения финансирования со стороны центров CDC, а также в условиях стресса, вызванного пандемией коронавирусной инфекции 2019 года (COVID-19).

**Методы:**

Авторы оценили следующие моменты: (i) продолжали ли национальные системы эпиднадзора за гриппом в странах, финансируемых центрами CDC, анализировать одно и то же количество образцов в период с 2013 по 2021 г.; (ii) участвовали ли эти системы в деятельности Глобальной системы Всемирной организации здравоохранения (ВОЗ) по эпиднадзору за гриппом и принятию ответных мер; (iii) протестировали ли эти системы достаточное количество образцов для выявления редких случаев заболевания или сигналов необычной активности; (iv) продемонстрировали ли эти системы стабильность до и во время пандемии коронавирусной инфекции 2019 года (COVID-19). Авторы использовали бюджетные документы центров CDC и данные из Глобальной системы ВОЗ по эпиднадзору за гриппом и принятию ответных мер.

**Результаты:**

Несмотря на то что центры CDC сократили финансирование на борьбу с гриппом в расчете на одну страну примерно на 75% за 10 лет, количество ежегодно тестируемых образцов оставалось стабильным (в среднем 2261). Отчетность существенно различалась в зависимости от страны и зоны передачи заболевания. На страны, финансируемые центрами CDC, приходился 71% (диапазон 61–75%) образцов, включенных в консультации ВОЗ по составу вакцин против вируса гриппа. В 2019 г. только восемь из 17 зон передачи вируса отправили достаточное количество образцов в сотрудничающие центры ВОЗ до проведения совещания по составу вакцины для надежной идентификации антигенных вариантов.

**Вывод:**

В глобальном понимании тенденций и сезонности гриппа достигнут значительный прогресс. Чтобы оптимизировать эпиднадзор для выявления атипичных вирусов гриппа и интегрировать молекулярное тестирование, секвенирование и отчетность о тяжелом остром респираторном синдроме коронавируса-2 в существующие системы, следует продолжить финансирование для поддержки этих усилий.

## Introduction

Global surveillance of influenza guides prevention and control decisions and monitors pandemic threats. Investment in global capacity-building for influenza surveillance was prompted by concerns about the pandemic potential of human infections with the highly pathogenic H5N1 avian influenza virus. As a result, dramatic improvements were made in testing capacity.^1^ While the coronavirus disease 2019 (COVID-19) pandemic highlighted the usefulness of strong disease surveillance systems, it strained insufficiently funded public health infrastructure and threatened the sustainability of surveillance systems.

In 2004, the Influenza Division of the United States Centers for Disease Control and Prevention (CDC) began support to health ministries to conduct influenza surveillance and improve pandemic preparedness using a funding model designed to gradually shift costs from donors to countries. These surveillance systems were critical during the 2009 H1N1 influenza pandemic response, and testing of respiratory specimens for influenza surged in the post-pandemic period.^2^ The Influenza Division’s early funding was intended to build sustainable capacity, with programmed reductions in its support after 10 years.^3^ Since 2013, 35 partner countries have transitioned to alternative funding sources, and by 2021, more than 70 countries had received some support from CDC for influenza surveillance.^2^

We aimed to assess the sustainability of the surveillance improvements made by countries supported by the Influenza Division as funding decreased. Of 64 partner countries continuing to receive funds from the Influenza Division in 2021, we assessed if their national influenza surveillance systems: (i) continued to analyse as many specimens as before funding decreases; (ii) participated in activities of the World Health Organization’s (WHO) Global Influenza Surveillance and Response System, e.g. national influenza centres reported to WHO FluNet (a global influenza surveillance reporting platform) and contributed specimens to WHO consultations on the composition of influenza virus vaccines; (iii) tested and shipped enough specimens to detect rare events or signals of unusual activity; and (iv) demonstrated stability both before the COVID-19 pandemic and when facing the stress associated with the pandemic, that is, if they maintained levels of testing and reporting consistent with pre-pandemic levels.

## Methods

### Funding to countries

We used budget records of the Influenza Division from 2013 to 2021 to explore relationships between CDC funding and surveillance activity. We consulted progress reports of WHO’s Pandemic Influenza Preparedness Framework, a global framework for pandemic influenza preparedness, for 2013–2021 to identify our partner countries that received additional external funding via that mechanism.^4^ To estimate changes in gross cost per specimen among partner countries funded for 10 years or more, we conducted a linear regression analysis between median annual award and annual median number of specimens reported to FluNet.

### FluNet participation and molecular testing

To evaluate the contribution of countries funded by the division to global influenza situational awareness, i.e. observation of circulating viruses, intensity of activity and identification of atypical activity, we calculated the proportion of Influenza Division partners among all countries reporting to FluNet. We reported the number of specimens tested and influenza-positive specimens per week reported to FluNet by partner countries aggregated by geographically contiguous areas with similar influenza transmission patterns (transmission zones).^5^

We explored increases in the volume of molecular testing reported to the Global Influenza Surveillance and Response System by partner countries throughout the CDC investment period using linear regression analysis. Finally, to assess the capacity of our partner countries to maintain surveillance during the COVID-19 pandemic, we compared the number of specimens tested annually, weekly and by epidemic period in 2019 and 2021.

### Advanced characterization of specimens

We explored the impact of the Influenza Division programme on the representativeness of data informing the biannual consultation to determine influenza vaccine composition and on global capacity to monitor the frequency and geographical diversity of genetic and antigenic change. We collected genetic and antigenic characterization and sequencing data from the WHO collaborating centre at CDC and combined these data with data uploaded to the EpiFlu^TM^ database (a global database of influenza genetic sequences) by all other collaborating centres for our partner countries. We used these data to assess the quantity and representativeness of genetic sequences collected worldwide.

We summed the number of specimens shared with WHO collaborating centres and submitted to the EpiFlu^TM^ database to detect temporal changes potentially associated with Influenza Division funding. We analysed submissions by transmission zone for the 3-month period before the vaccine composition meeting to explore geographical representativeness of decisions on vaccine selection.

To identify transmission zones that produce the greatest number of atypical (i.e. non-endemic) viruses, we analysed the frequency of sequences from viruses characterized as antigenic drift variants (i.e. low reactors) submitted to the WHO collaborating centre at CDC or uploaded to the EpiFlu^TM^ database by other collaborating centres. We compared these numbers across transmission zones to identify regions that shared the most atypical viruses. We analysed the relationship between the mean number of influenza-positive viruses reported to FluNet and the mean number of viruses sequenced for each transmission zone to identify the proportion of positive viruses sequenced.

Using 2019 World Bank population estimates for partner countries of the Influenza Division, we calculated the population proportion in each transmission zone and determined the expected number of specimen submissions for each zone if distributed proportionally to population. We calculated the difference between the actual and expected submissions to evaluate population-based representativeness.

## Results

### Funding to countries

**Fig. 1:**
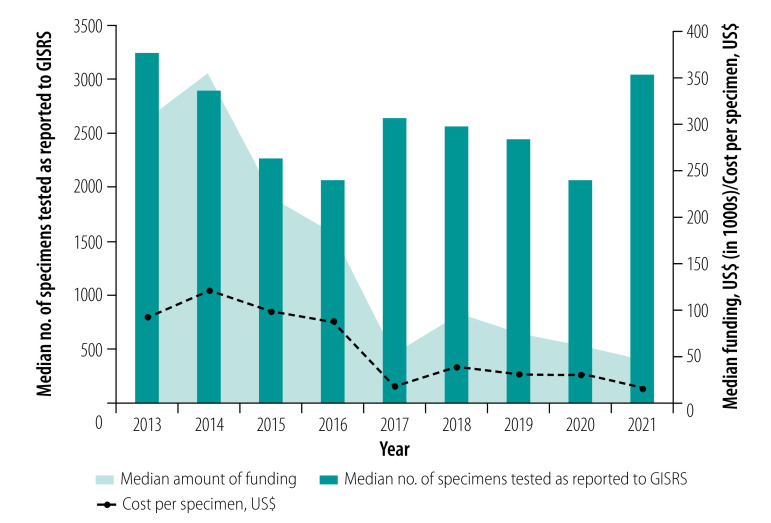
Number of specimens tested in countries supported by the Centers for Disease Control and Prevention, total funding and cost to CDC per specimen, 2013–2021

CDC: Centers for Disease Control and Prevention; GISRS: Global Influenza Surveillance and Response System; US$: United States dollars.

Note: *P* = 0.002 for decrease in cost per specimen, 2013–2021.

During 2013–2021, the Influenza Division directly or indirectly funded 70 countries, which had about 70% of the 2021 world population. We analysed data for 64 countries receiving funding before the COVID-19 pandemic, i.e. as of 2019. In 2021, there were 40 funded agreements. Six (15%) agreements had been in place for 1–5 years, five (12%) for 6–10 years and 29 (73%) for more than 10 years. Of the 34 countries that had received 10 years of funding by 2021, the median award was 300 000 United States dollars (US$; interquartile range, IQR: 282 500–400 000) in 2013 and US$ 50 000 (IQR: 24 981–100 000) in 2021 (Fig. 1). Nearly half (48%; 31/64) of the Influenza Division partner countries received at least 1 year of funding from the WHO Pandemic Influenza Preparedness Framework between 2013 and 2021.^4^

### FluNet participation

**Fig. 2:**
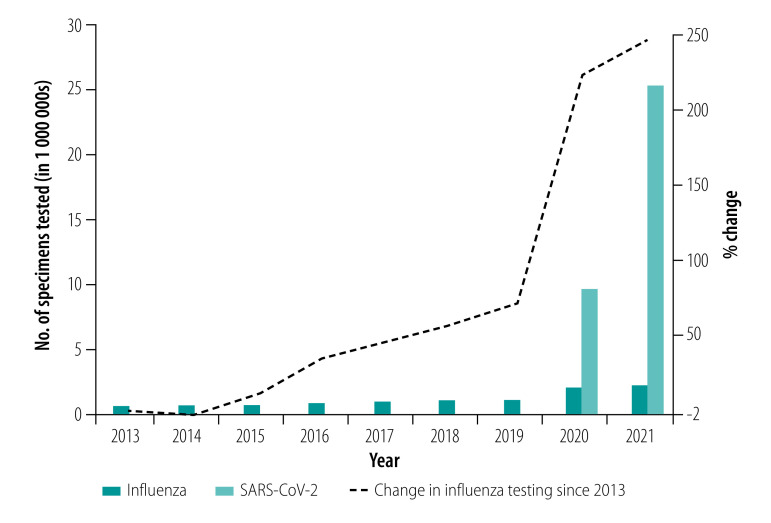
Molecular testing volume in countries supported by the Centers for Disease Control and Prevention, 2013–2021

**Fig. 3:**
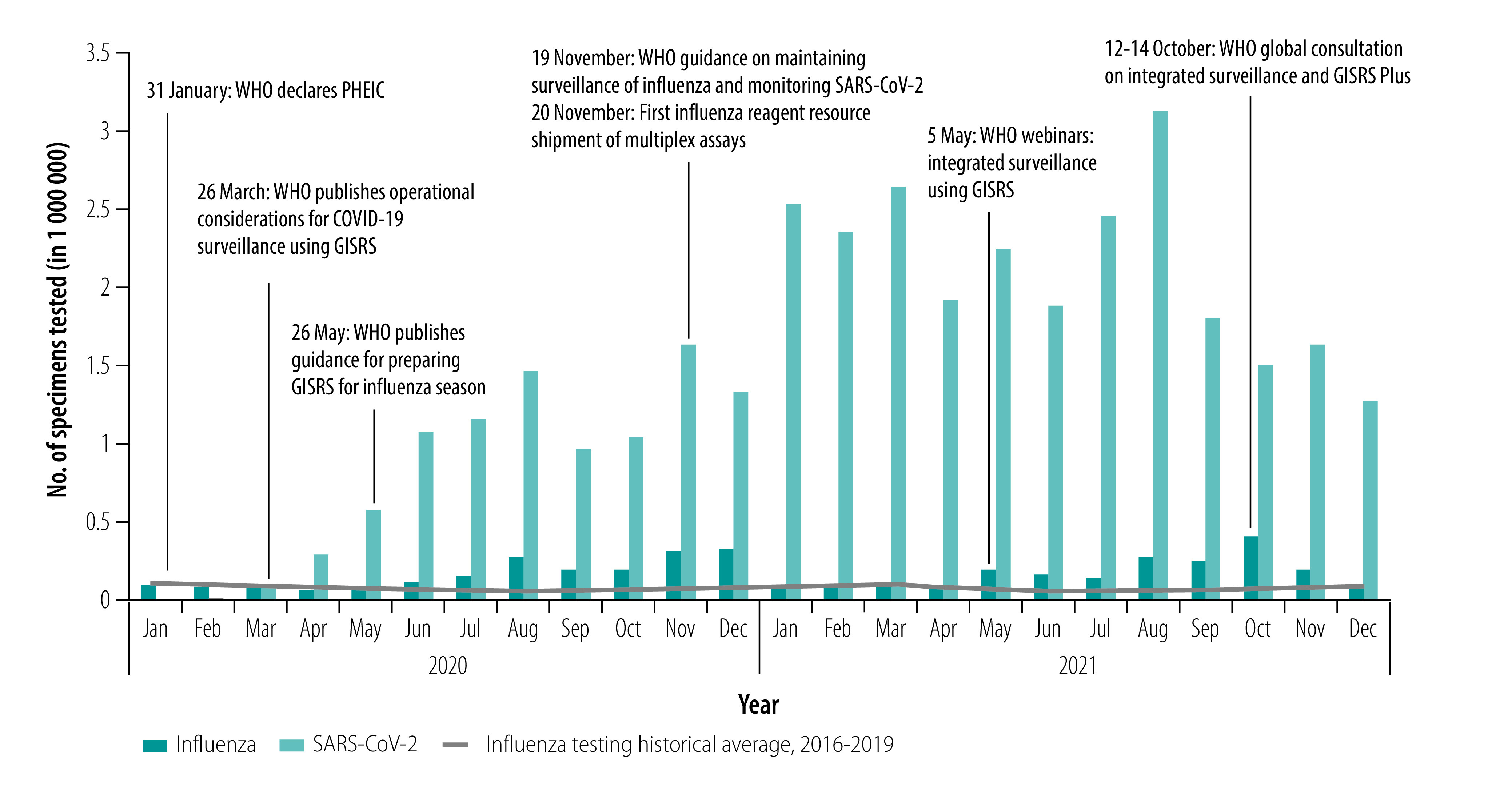
Number of specimens tested for influenza and SARS-CoV-2 molecular testing in countries supported by the Centers for Disease Control and Prevention, by month, 2020–2021

COVID-19: coronavirus disease 2019; GISRS: Global Influenza Surveillance and Response System; PHEIC: public health emergency of international concern; SARS-CoV-2: severe acute respiratory syndrome coronavirus 2; WHO: World Health Organization.

The 64 countries in our analysis represented 63% of the 102 WHO Member States reporting data to FluNet in 2021. While the weekly number of specimens tested was similar during 2021 (33; IQR: 11–86) compared with 2019 (35; IQR: 14–90), influenza detections were lower in 2021 (2; IQR: 0–10) than in 2019 (median 4; IQR: 0–17). We observed similar differences in pre- and peri-pandemic testing during epidemic periods (periods of sustained activity above baseline): a median of 4592 (IQR: 1669–18 574) tests and 58 (IQR: 8–512) influenza-positive results were reported to FluNet per epidemic period from each WHO transmission zone included in our analysis in 2021 compared with a median of 5529 (IQR: 1142–13 369) tests and 1355 (IQR: 366–2761) influenza-positive results in 2019. The annual average number of specimens tested and reported to FluNet increased linearly between 2013 and 2021 at a rate of almost 200 000 specimens a year, a statistically significant increase (*P*-value 0.002; Fig. 2). In 2020 and 2021, the volume of influenza testing varied monthly, but followed a similar pattern to severe acute respiratory syndrome coronavirus 2 (SARS-CoV-2) testing (Fig. 3). For most months in 2020 and 2021, influenza testing was higher than the historical monthly average of influenza testing for 2016–2019.

SARS-CoV-2: severe acute respiratory syndrome coronavirus 2.

Note: *P* = 0.002 for increase in average number of specimens tested, 2013–2021.

Of the 35 countries that had received 10 years of funding by 2021, the median number of specimens tested was 3247 (IQR: 1312–5042) in 2013 and 3051 (IQR: 892–10 344) in 2021. When comparing the median award with the median number of specimens tested, we saw a linear decrease in cost per specimen from US$ 92 in 2013 to US$ 16 in 2021 (*P*-value 0.002; Fig. 1).

### Advanced characterization of specimens

The number of partner countries that shipped specimens to a WHO collaborating centre significantly increased from 44 (69% of 64) in 2013 to 56 (88%) in 2019 (*P*-value 0.02). Shipments from partner countries of the Influenza Division accounted for an average of 71% (range: 61–94%) of specimens included in the genetic sequencing data package submitted to the vaccine composition meeting; partner countries made up 81% (1797/2222) and 94% (4765/5078) of submissions during the February and September 2021 vaccine composition meetings, respectively. This change represented an increase of more than 100% in the annual number of specimens shipped by partner countries, from 14 956 in 2013 to 36 868 in 2019; this number fell during the low circulation of influenza during the peri-pandemic period.^6^ Partner countries accounted for an average of 44% (range 39–47) of all sequences of human seasonal influenza viruses uploaded to EpiFlu^TM^ by any WHO collaborating centre between 2013 and 2019.

During 2013–2019, 8.3% (460/5518) of influenza A(H1N1), 21% (1057/5070) of influenza A(H3N2), 16% (370/2373) of influenza B Victoria and 15% (401/2673) of influenza B Yamagata haemagglutinin sequences were classified into clades with a prevalence of less than 10%. Similarly, of 14 059 influenza viruses that were antigenically characterized, 521 (4%) were antigenic drift variants which did not react strongly in laboratory assays (data repository).^6^ In 2013–2019, 2279 specimens were identified as rare genetic clades (i.e. clades comprising less than 10% of clades identified in genetic sequencing). These 2279 clades were most often identified in Eastern Asia (479; 21%), South East Asia (340; 15%) and Eastern Europe (340; 15%; *P*-value 0.0001; data repository).^6^ Of the 521 drift variants identified in 2013–2021, most were identified in specimens shipped from South East Asia (145; 28%) followed by Southern Asia (101; 19%) and Eastern Europe (47; 9%; *P*-value 0.0001; data repository).^6^

### Geographical representativeness of funded countries

Reporting varied substantially by country and transmission zone. In 2019, the total population of partner countries was 5 453 110 000. Eastern Asia contained 26% (1 400 520 000) of that population but accounted for 69% (680 186) of the average number of specimens tested per year for 2016–2019 by all partners (991 219), as reported to FluNet, and 87% of specimens shipped to collaborating centres. Tropical South America accounted for 2% (17 338/991 219) of the specimens reported to FluNet, but 5% (243 560 000) of the population of partner countries. In contrast, Southern Asia had 34% (1 835 731 000) of the population of partner countries but accounted for only 4% (40 718) of specimens tested, as reported to FluNet (data repository).^6^ The greatest proportions of the 1067 specimens included in the February 2019 vaccine composition meeting package were from Eastern Asia (250; 23%), South East Asia (248; 23%) and Eastern Europe (155; 15%). Only 27% (292/1067) of the remaining specimens came from zones with 61% of the northern hemisphere population. Similarly, Eastern Asia (399; 23%), Eastern Europe (230; 13%) and Central America and the Caribbean (198; 12%) accounted for the greatest proportion of the 1717 specimens in the package for the September 2019 vaccine composition meeting. Only 21% (366/1717) of the specimens in the package originated from other southern hemisphere transmission zones, which typically have peak influenza activity in May–September. Overall, 3% (IQR: 1–8) of the positive viruses reported to FluNet were reported to EpiFlu^TM^ as having been sequenced annually. However, this figure varied greatly by transmission zone, with little change from 2013 to 2019. Oceania Melanesia and Polynesia, and Northern Africa, respectively, sequenced a mean of two and six influenza viruses annually, while Central America, Southern Asia and South East Asia sequenced an average of 289, 255 and 252 viruses, respectively, each year.

### Ability to identify rare variants

Before the COVID-19 pandemic, 41 of 48 (85%) countries with > 5 years of data tested enough samples in 2021 to define their epidemic period (required sample size ≥ 100 specimens).^7^ Two (12%) of the 17 transmission zones tested enough specimens by molecular testing each week to reliably identify atypical viruses circulating at a hypothetical prevalence of 0.1% (required sample size ≥ 3838 specimens; Table 1). Eleven of the 17 transmission zones tested enough specimens over the epidemic period to reliably identify atypical viruses. In 2019, only eight of the 17 transmission zones shipped enough specimens to WHO collaborating centres in the 3 months before the vaccine composition meeting to reliably identify antigenic variants and rare haemagglutinin genetic clades at a hypothetical prevalence of 3.7% per epidemic period (required sample size ≥ 100 specimens; Table 1). The most underpowered transmission zones for detecting antigenic variants and rare haemagglutinin genetic clades were Middle Africa, Oceania Melanesia and Polynesia, and Central Asia. Of note, all transmission zones except Middle Africa, and Oceania Melanesia and Polynesia tested enough samples to identify hypothetical antigenic drift variants if all influenza-positive samples identified through reverse transcription polymerase chain reaction testing were further characterized.

**Table 1:** Samples tested, identified and submitted within the influenza surveillance, by transmission zone, 2013–2021

Influenza transmission zone	WHO characterized epidemic months	Mean no. of samples tested,^a^ 2013–2021		Mean no. of influenza-positive samples^a^ per epidemic, 2013–2021	Antigenic drift variants identified 2013–2021, total no. (%)	Samples sent to collaborating centres in 3 months before Sep 2019 vaccine composition meeting, mean no. (%)	Samples sent to collaborating centres in 3 months before Feb 2019 vaccine composition meeting, mean no. (%)	Population-based deficit or surplus in sample submissions in 2019, %
		Week	Epidemic period					
Eastern Africa	Dec–Jan	225	1 553	217	57 (11)	86 (3)	121 (2)	−6
Northern Africa	Dec–Feb	223	4 809	1 149	1 (0)	10 (0)	55 (1)	−2
Middle Africa	Dec–May	27	761	87	4 (1)	17 (1)	29 (0)	−1
Western Africa	Sep–Mar	186	5 891	833	28 (5)	68 (2)	106 (1)	−4
Southern Africa	May–Sep	144	4 221	788	2 (0)	65 (2)	28 (0)	−1
Central America and Caribbean	Jun–Oct	337	8 257	888	22 (4)	105 (3)	89 (1)	0
Temperate South America	Jun–Aug	166	3 181	570	7 (1)	28 (1)	20 (0)	0
Tropical South America	May–Sep	5 137	135 635	1 409	47 (9)	179 (6)	66 (1)	−4
North America	Dec–Mar	601	16 032	3 587	26 (5)	30 (1)	31 (0)	−2
Eastern Asia	Jan–Mar	10 952	176 237	36 441	115 (22)	1 910 (60)	6 324 (84)	63^b^
Central Asia	Dec–Feb	14	502	151	0 (0)	31 (1)	27 (0)	0
Western Asia	Dec–Mar	36	1 074	352	1 (0)	24 (1)	33 (0)	0
Southern Asia	Dec–Apr	895	22 260	3 530	37 (7)	133 (4)	111 (1)	–32^c^
Eastern Europe	Jan–Apr	2 582	76 268	17 262	38 (7)	201 (6)	97 (1)	−3
South West Europe	Dec–Mar	191	6 525	2 512	3 (1)	69 (2)	57 (1)	0
Oceania Melanesia and Polynesia	Jul–Sep	31	475	92	0 (0)	78 (2)	69 (1)	0
South East Asia	Jul–Oct	315	5 976	1 106	131 (25)	176 (5)	259 (3)	−8
**Total**	**NA**	**NA**	**NA**	**NA**	**519**	**3210**	**7 522**	**NA**

NA: not applicable; WHO: World Health Organization.

^a^ Identified by polymerase chain reaction test.

^b^ Over-represented (more specimens than proportional to the population).

^c^ Under-represented (fewer specimens than proportional to the population).

Notes: Tropical South America and Eastern Asia tested enough specimens each week to reliably identify atypical viruses. Central America and Caribbean, Tropical South America, Eastern Asia, Eastern Europe, South West Europe, Southern Asia, Northern Africa, Western Africa, Southern Africa, North America and South East Asia tested enough specimens over the epidemic period to reliably identify atypical viruses. Central America and Caribbean, Tropical South America, Eastern Asia, Eastern Europe, Southern Asia, Eastern Africa, Western Africa and South East Asia sent enough specimens to WHO collaborating centres to reliably detect viruses or rare variants or clades that could not be subtyped.

## Discussion

Our findings suggest that Influenza Division investments established sustainable programmes, with a linear decrease in costs to the division per influenza specimen tested. The transition from external to domestic funding was implicit in the funding model and was intended to foster country ownership and investment by local stakeholders, similar to models used by other global health organizations.^8^^,^^9^ Partner countries tested and reported a similar number of specimens before and after transitioning from Influenza Division funding to other funding sources,^10^ with a surge during the COVID-19 pandemic. These partners continued to participate meaningfully and improve their contributions to the Global Influenza Surveillance and Response System during 2013–2021 despite an average programmed funding decrease. While in some countries additional donors supported influenza surveillance, to our knowledge their contributions have been modest and often focused on research or have been awarded to nongovernment partners.^11^,^12^ It is possible that a surge in donor funds during the COVID-19 pandemic facilitated expansion in testing capacity after 2020.

The Global Influenza Surveillance and Response System provides critical information for selection of influenza vaccine strains and surveillance for new viruses. We observed that influenza testing volumes reported to FluNet by partner countries were 3.5 times higher in 2021 than in 2013, and the number of specimens submitted to WHO collaborating centres from 2013 to 2019 nearly doubled, which contributed to an overall strengthening of the Global Influenza Surveillance and Response System.^13^ The volume of molecular influenza testing during the COVID-19 peri-pandemic period was 1.5 times higher in 2021 than in 2019. The increase in the number and geographical breadth of specimens expanded genetic sequencing and identification of antigenic variants, which likely improved the representativeness of influenza vaccine strains. This larger pool of specimens available for sequencing potentially increases global capacity to identify rare viruses.

Information generated by influenza surveillance systems facilitates evidence-based influenza prevention and control policies and programmes. Before the expansion of global testing and reporting, little was known about the circulation of influenza viruses and the burden of influenza disease in tropical and subtropical areas.^14^ During our analytic time period (2013–2021), 36 partner countries of the Influenza Division published national estimates of the burden of influenza disease or were included in regional estimates, and these estimates were used to justify investments in expanding or introducing new vaccination programmes.^15^^–^^18^ These estimates have also been used to plan the timing of national influenza vaccination campaigns.^7^

Sustained gains among Influenza Division partner countries through the COVID-19 pandemic suggest commitments to conduct national surveillance despite the challenge of a stressed system and other challenges, such as political will to replace donor funding or inaccurate estimates of financial needs.^19^ Continuity of influenza testing during the pandemic may have been influenced by WHO guidance^20^^,^^21^ and webinars about the integration of SARS-CoV-2 testing using the Global Influenza Surveillance and Response System. Reminders to continue influenza testing in advance of the typical influenza seasons might have contributed to consistent testing for influenza in addition to SARS-CoV-2 during the peri-pandemic period.

The magnitude of surveillance gains has not been geographically homogeneous. These differences may represent missed opportunities to rapidly identify rare events that could be the first signal of a public health emergency of international concern and identify emerging viruses that could become seasonal or pandemic influenza vaccines.^22^ The Eastern Asia transmission zone consistently tested enough specimens to detect a rare event weekly, while in the remaining zones, testing was sufficient only when specimens were aggregated during a 3-month influenza epidemic period. The Southern Asia transmission zone, home to 27% of the global population, provided only a small portion of the total specimens reported to FluNet. While 11 of 17 transmission zones were able to identify non-endemic viruses circulating at a low prevalence (e.g. < 0.1%) during their three-peak epidemic months, an insufficient number of specimens were collected during epidemic periods in other zones. Given country-level testing disparities, it may be sensible to aggregate data from epidemic zones to identify rare events. We have identified transmission zones that would benefit most from technical assistance and guidance on identifying atypical events, the target sample sizes needed to do so and the appropriate time frames. The mismatch in population size to specimen volume may indicate regions with resource-constrained health systems that require greater financial and technical investment and an increase in political interest in surveillance of respiratory viruses and mitigation of their disease burden.

The COVID-19 pandemic tested the pandemic preparedness capacity that the Influenza Division programme intended to build. Partner countries met this challenge by quickly operationalizing their influenza surveillance systems to detect and monitor SARS-CoV-2 activity.^23^ With similar case definitions^10^^,^^24^^,^^25^ and molecular testing and reporting platforms, the surveillance systems supported by the Influenza Division proved agile enough to monitor other respiratory viruses. The COVID-19 pandemic led to substantial increases in testing by national influenza centres – the SARS-CoV-2 testing volume in 2021 was 23 times greater than the 2019 influenza testing volume. COVID-19 response investments are being used so that national influenza centres can routinely test respiratory specimens for multiple viral pathogens, employ new assays, and sequence and improve informatics platforms, thereby enhancing broader respiratory disease surveillance activities globally.

Our evaluation was subject to several limitations. We relied on publicly available data, which might not represent the entirety of each country’s influenza surveillance data, nor differentiate between routine surveillance and outbreak-related testing data. Reporting to the Global Influenza Surveillance and Response System and EpiFlu^TM^ and shipment of specimens to collaborating centres are voluntary, and not all countries share complete information about their activities. As a result, our estimates for power to detect unusual events, proportional representation and overall gains might be biased. We present our analyses using theoretical thresholds to consider potential sample size requirements to detect rare events; we are unaware of a consensus about standard influenza testing sample sizes, thresholds or triggers for public health action. Our analyses included data from Influenza Division partner countries only and do not represent comprehensive global estimates. While our partner countries are worldwide, they do not include high-income countries that typically have well developed influenza surveillance systems. We believe, however, that gains in global surveillance have primarily been in low- and middle-income countries that have expanded their surveillance since donors began funding surveillance in 2004. Likewise, advances in surveillance in tropical and subtropical regions may afford new opportunities to identify emerging viruses and to identify viruses that might start epidemics in temperate regions.^26^^,^^27^ While we believe gains are partially attributable to Influenza Division investments, we cannot quantify the contributions of other donors. We recognize that China has an extensive surveillance network and some of our analyses may be biased by the amount of data from there. Similarly, Brazil expanded surveillance during the COVID-19 pandemic, which may also bias our estimates.

In conclusion, investment in infrastructure led to sustained growth in surveillance capacity among partner countries. The initial 10-year investment in capacity-building yielded gains that, as of 2021, proved sustainable despite decreases in Influenza Division funding and the stress of a respiratory disease pandemic. Partner surveillance systems demonstrated agility in integrating a non-influenza pathogen into routine surveillance within the Global Influenza Surveillance and Response System.^28^ While global gains in surveillance have been substantial, groups of neighbouring countries within transmission zones, rather than individual countries, are testing enough specimens to reliably detect unusual events. Strategic improvements, such as increasing capacity to perform next-generation sequencing within transmission zones, may provide opportunities to improve global situational awareness of influenza activity trends and the emergence of atypical viruses.

## References

[1] Johnson LE, Clará W, Gambhir M, Chacón-Fuentes R, Marín-Correa C, Jara J, et al. Improvements in pandemic preparedness in 8 Central American countries, 2008-2012. BMC Health Serv Res. 2014 May 9;14(1):209. 10.1186/1472-6963-14-209 24886275 PMC4022548

[2] Polansky LS, Outin-Blenman S, Moen AC. Improved global capacity for influenza surveillance. Emerg Infect Dis. 2016 Jun;22(6):993–1001. 10.3201/eid2206.151521 27192395 PMC4880096

[3] Kennedy POS, Rodriguez TR, Polansky L, McCarron M, Siener KR, Moen AC. Building surveillance capacity: lessons learned from a ten year experience. J Infect Dis Epidemiol. 2017;3(1). 10.23937/2474-3658/1510026

[4] Pandemic Influenza Preparedness Framework partnership contribution [internet]. Geneva: World Health Organization; 2022. Available from: https://www.who.int/initiatives/pandemic-influenza-preparedness-framework/partnership-contribution [cited 2022 Feb 27].

[5] Transmission zones. Geneva: World Health Organization; 2015. Available from: https://cdn.who.int/media/docs/default-source/influenza/influenza-updates/2020/influenza_transmission_zones20180914.pdf?sfvrsn=dba8eca5_3 [cited 2021 Jan 25].

[6] McCarron M, Kondor R, Zureick K, Griffin C, Fuster C, Hammond A, et al. Stability of improvements in influenza surveillance, 2013–2021 Supplementary materials. London: figshare; 2022 10.6084/m9.figshare.19526182. 10.6084/m9.figshare.19526182

[7] Hirve S, Newman LP, Paget J, Azziz-Baumgartner E, Fitzner J, Bhat N, et al. Influenza seasonality in the tropics and subtropics – when to vaccinate? PLoS One. 2016 Apr 27;11(4):e0153003. 10.1371/journal.pone.0153003 27119988 PMC4847850

[8] Eligibility and transition policy [internet]. Geneva: Gavi, the Vaccine Alliance; 2020. Available from: https://www.gavi.org/programmes-impact/programmatic-policies/eligibility-and-transitioning-policy [2020 Mar 26].

[9] Sustainability, transition & co-financing [internet]. Geneva: Global Fund; 2019. Available from: https://www.theglobalfund.org/en/sustainability-transition-and-co-financing/ [cited 2020 Mar 26].

[10] Global surveillance for COVID-19 caused by human infection with COVID-19 virus. Interim guidance, 20 March 2020. Geneva: World Health Organization; 2020. Available from: https://apps.who.int/iris/bitstream/handle/10665/331506/WHO-2019-nCoV-SurveillanceGuidance-2020.6-eng.pdf [cited 2022 Feb 27].

[11] Igboh LS, McMorrow M, Tempia S, Emukule GO, Talla Nzussouo N, McCarron M, et al.; ANISE Network Working Group. Influenza surveillance capacity improvements in Africa during 2011–2017. Influenza Other Respir Viruses. 2021 Jul;15(4):495–505. 10.1111/irv.12818 33150650 PMC8189239

[12] USA spending [internet]. Washington, DC: US Office of Management and Budget; 2022. Available from: https://www.usaspending.gov/ [cited 2022 Jan 3].

[13] Ampofo WK, Azziz-Baumgartner E, Bashir U, Cox NJ, Fasce R, Giovanni M, et al.; WHO Writing Group. Strengthening the influenza vaccine virus selection and development process: Report of the 3rd WHO informal consultation for improving influenza vaccine virus selection held at WHO headquarters, Geneva, Switzerland, 1–3 April 2014. Vaccine. 2015 Aug 26;33(36):4368–82. 10.1016/j.vaccine.2015.06.090 26148877

[14] Durand LO, Cheng PY, Palekar R, Clara W, Jara J, Cerpa M, et al. Timing of influenza epidemics and vaccines in the American tropics, 2002–2008, 2011–2014. Influenza Other Respir Viruses. 2016 May;10(3):170–5. 10.1111/irv.12371 26701079 PMC4814866

[15] Monto AS. Reflections on the Global Influenza Surveillance and Response System (GISRS) at 65 years: an expanding framework for influenza detection, prevention and control. Influenza Other Respir Viruses. 2018 Jan;12(1):10–2. 10.1111/irv.12511 29460424 PMC5818347

[16] Hirve S, Lambach P, Paget J, Vandemaele K, Fitzner J, Zhang W. Seasonal influenza vaccine policy, use and effectiveness in the tropics and subtropics – a systematic literature review. Influenza Other Respir Viruses. 2016 Jul;10(4):254–67. 10.1111/irv.12374 26842617 PMC4910173

[17] Newman LP, Bhat N, Fleming JA, Neuzil KM. Global influenza seasonality to inform country-level vaccine programs: an analysis of WHO FluNet influenza surveillance data between 2011 and 2016. PLoS One. 2018 Feb 21;13(2):e0193263. 10.1371/journal.pone.0193263 29466459 PMC5821378

[18] Ropero-Alvarez AM, Kurtis HJ, Danovaro-Holliday MC, Ruiz-Matus C, Andrus JK. Expansion of seasonal influenza vaccination in the Americas. BMC Public Health. 2009 Sep 24;9(1):361. 10.1186/1471-2458-9-361 19778430 PMC2764707

[19] Gotsadze G, Chikovani I, Sulaberidze L, Gotsadze T, Goguadze K, Tavanxhi N. The challenges of transition from donor-funded programs: results from a theory-driven multi-country comparative case study of programs in Eastern Europe and Central Asia supported by the Global Fund. Glob Health Sci Pract. 2019 Jun 27;7(2):258–72. 10.9745/GHSP-D-18-00425 31249022 PMC6641812

[20] Operational considerations for COVID-19 surveillance using GISRS: interim guidance. Geneva: World Health Organization; 2020. Available from: https://www.who.int/publications/i/item/operational-considerations-for-covid-19-surveillance-using-gisrs-interim-guidance [2022 Feb 27].

[21] Maintaining surveillance of influenza and monitoring SARS-CoV-2 – adapting Global Influenza Surveillance and Response System (GISRS) and sentinel systems during the COVID-19 pandemic. Interim guidance. Geneva: World Health Organization; 2020. Available from: https://apps.who.int/iris/handle/10665/336689 [cited 2022 Feb 27].

[22] Braden CR, Dowell SF, Jernigan DB, Hughes JM. Progress in global surveillance and response capacity 10 years after severe acute respiratory syndrome. Emerg Infect Dis. 2013 Jun;19(6):864–9. 10.3201/eid1906.130192 23731871 PMC3713843

[23] Marcenac P, McCarron M, Davis W, Igboh LS, Mott JA, Lafond KE, et al. Leveraging international influenza surveillance systems and programs during the COVID-19 pandemic. Atlanta: US Centers for Disease Control and Prevention; 2021. 10.3201/eid2813.212248 PMC9745234 36502434

[24] WHO surveillance case definitions for ILI and SARI [internet]. Geneva: World Health Organization; 2020. Available from: https://www.who.int/teams/global-influenza-programme/surveillance-and-monitoring/case-definitions-for-ili-and-sari [cited 2020 Jan 17].

[25] Middle East respiratory syndrome coronavirus: case definition for reporting to WHO. Geneva: World Health Organization; 2017. Available from: https://www.who.int/csr/disease/coronavirus_infections/mers-interim-case-definition.pdf?ua=1 [cited 2020 Jan 17].

[26] Rambaut A, Pybus OG, Nelson MI, Viboud C, Taubenberger JK, Holmes EC. The genomic and epidemiological dynamics of human influenza A virus. Nature. 2008 May 29;453(7195):615–9. 10.1038/nature06945 18418375 PMC2441973

[27] Viboud C, Alonso WJ, Simonsen L. Influenza in tropical regions. PLoS Med. 2006 Apr;3(4):e89. 10.1371/journal.pmed.0030089 16509764 PMC1391975

[28] End-to-end integration of SARS-CoV-2 and influenza sentinel surveillance: revised interim guidance. Geneva: World Health Organization; 2022. Available from: https://apps.who.int/iris/handle/10665/351409 [cited 2022 Feb 27].

